# Machine learning models to predict in-hospital mortality in septic patients with diabetes

**DOI:** 10.3389/fendo.2022.1034251

**Published:** 2022-11-16

**Authors:** Jing Qi, Jingchao Lei, Nanyi Li, Dan Huang, Huaizheng Liu, Kefu Zhou, Zheren Dai, Chuanzheng Sun

**Affiliations:** Department of Emergency, Third Xiangya Hospital of Central South University, Changsha, China

**Keywords:** machine learning, sepsis, diabetes, in-hospital mortality, risk factors

## Abstract

**Background:**

Sepsis is a leading cause of morbidity and mortality in hospitalized patients. Up to now, there are no well-established longitudinal networks from molecular mechanisms to clinical phenotypes in sepsis. Adding to the problem, about one of the five patients presented with diabetes. For this subgroup, management is difficult, and prognosis is difficult to evaluate.

**Methods:**

From the three databases, a total of 7,001 patients were enrolled on the basis of sepsis-3 standard and diabetes diagnosis. Input variable selection is based on the result of correlation analysis in a handpicking way, and 53 variables were left. A total of 5,727 records were collected from Medical Information Mart for Intensive Care database and randomly split into a training set and an internal validation set at a ratio of 7:3. Then, logistic regression with lasso regularization, Bayes logistic regression, decision tree, random forest, and XGBoost were conducted to build the predictive model by using training set. Then, the models were tested by the internal validation set. The data from eICU Collaborative Research Database (n = 815) and dtChina critical care database (n = 459) were used to test the model performance as the external validation set.

**Results:**

In the internal validation set, the accuracy values of logistic regression with lasso regularization, Bayes logistic regression, decision tree, random forest, and XGBoost were 0.878, 0.883, 0.865, 0.883, and 0.882, respectively. Likewise, in the external validation set 1, lasso regularization = 0.879, Bayes logistic regression = 0.877, decision tree = 0.865, random forest = 0.886, and XGBoost = 0.875. In the external validation set 2, lasso regularization = 0.715, Bayes logistic regression = 0.745, decision tree = 0.763, random forest = 0.760, and XGBoost = 0.699.

**Conclusion:**

The top three models for internal validation set were Bayes logistic regression, random forest, and XGBoost, whereas the top three models for external validation set 1 were random forest, logistic regression, and Bayes logistic regression. In addition, the top three models for the external validation set 2 were decision tree, random forest, and Bayes logistic regression. Random forest model performed well with the training and three validation sets. The most important features are age, albumin, and lactate.

## 1 Introduction

The definition of sepsis has updated to sepsis-3 (1), namely, a life-threatening dysfunction caused by a dysregulated host response to the infection, in 2016, which indicates huge workload in related fields. On the other hand, our understanding of this disease is limited given the generalized definition and the incidence and mortality rate that still remained high (2). From 1979 to 2015, hospital mortality was even 17% and 26% for sepsis and severe sepsis, respectively, in case of excluding the data from lower-income countries (3). Up to now, we have not established longitudinal networks from molecular mechanisms to heterogeneous clinical phenotypes. Together with the related influence elements of the infection, individual host heterogeneity makes the whole disease network vague. To figure it out, establishing criteria to distinguish subgroup of sepsis has been proposed (4).

Attention should be paid to patients with sepsis presented with diabetes, which account for 20% (5, 6). The prevalence of diabetes is surging, due to the improvement of the living standards and spreading of Western lifestyle. The number of people with diabetes rose from 108 million in 1980 to 422 million in 2014 (7). As a major cause of blindness, kidney failure, heart attacks, stroke, and lower limb amputation, diabetes was the ninth leading cause (8) of death with an estimated 1.5 million deaths per year directly caused by it (7). Obviously, diabetes has been a non-negligible global healthy burden for a long time. Moreover, the complicated pathogenesis and multi-organ complications make it difficult for clinicians to evaluate the prognosis, especially when the sepsis presented. Many cohort studies had published with the purpose to explore the interaction between the blood sugar level and infection or diabetes and sepsis. However, the conclusions do not show a high degree of conformance.

Therefore, to build an efficient clinical tool, five machine learning models were established to predict the in-hospital mortality of patients with sepsis with diabetes. The accuracy was compared to distinguish the performance, and important features were discussed to get hints for guiding clinical practice.

## 2 Materials and methods

### 2.1 Data sources and cohort selection

Data were collected from two of the largest critical care databases in USA, the Medical Information Mart for Intensive Care (MIMIC-IV) database (9, 10) and the eICU Collaborative Research Database (eICU-CRD, version 2.0) (11), and a large critical care database in China (named “dtChina”) (12). This study was approved by the Institutional Review Broad (IRB) of the Massachusetts Institute of Technology (MIT) online (Record ID: 38889441), and informed consent was waived. All data were deidentified for privacy protection and extracted by Structured Query Language with PostgreSQL 9.6 as described in a previous study (13). The study was reported in accordance with REporting of studies Conducted using Observational Routinely collected health Data (RECORD) statement (14).

**Figure 1:**
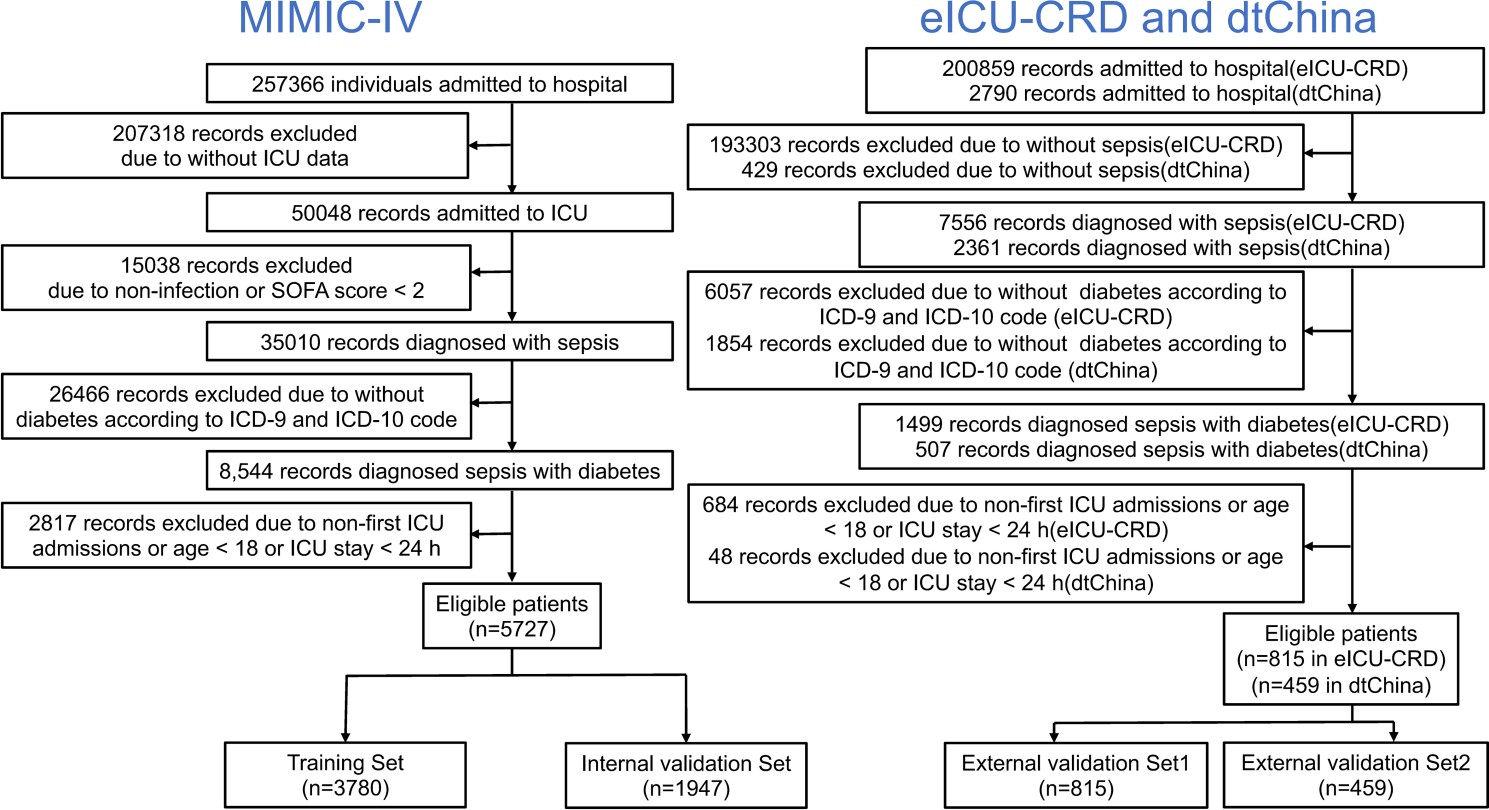
Flow chart depicting number of patients who were included in analysis after exclusion criteria. A total of 7001 records were enrolled.

The eligibility criteria in this study included the following: (1) patients were diagnosed as sepsis following the definition of Sepsis 3.0 (1); (2) patients were diagnosed with diabetes as comorbidity; (3) age ≥18 years old; and (4) lengths of intensive care unit (ICU) stays ≥ 1 day. For patients who were admitted to the ICU more than once, only the first ICU stay was considered, and patients were excluded with no ICU admission. Variables with over 30% missing values were excluded. The data were then randomly divided into a training set (66% of the data) and an internal validation set (remaining 34% of the data); finally, two large datasets including American and Chinese patients’ records were conducted as external validation sets (named Validation Set 1 and Validation Set 2, respectively). A summary of the study methods is shown in **Figure 1**.

### 2.2 Data collection and definition

Demographic data were collected including age and gender. Otherwise, the history of myocardial infarction, congestive heart failure, peripheral vascular disease, cerebrovascular disease, chronic pulmonary disease, peptic ulcer disease, paraplegia, chronic kidney disease, malignant cancer, and metastatic solid tumor were collected as comorbidities. The first record of vital sign was collected while admitted to ICU, including the heart rate, respiratory rate, systolic blood pressure, diastolic blood pressure, mean blood pressure, and oxygen saturation. The first record of laboratory data was collected within 72 h after admission to ICU, including white blood cell, neutrophils, lymphocytes, monocytes, eosinophils, basophils, red blood cell, platelet, hematocrit, hemoglobin, mean corpuscular hemoglobin (MCH), MCH concentration (MCHC), mean corpuscular volume (MCV), red cell distribution width (RDW), alanine transaminase (ALT), aspartate transaminase (AST), alkaline phosphatase (ALP), albumin, total bilirubin, blood urea nitrogen (BUN), blood creatinine, prothrombin time (PT), international normalized ratio (INR), blood sodium, potassium, calcium, chloride, bicarbonate, anion gap (AG), blood glucose, and lactate. The interventions included continuous renal replacement therapy (CRRT), ventilation, and vasopressor use (including dobutamine, dopamine, epinephrine, norepinephrine, and phenylephrine use). Data with more than 30% missing data were removed, and in-hospital mortality was used as outcome of all patients.

### 2.3 Statistical analysis

The counting data are shown as percentages, and measurement data are shown as the mean or median with standard deviation (SD). The chi-squared test or Fisher’s exact test was used to evaluate the comparison of counting data, the Student’s t-test or one-way ANOVA was used for the comparison of continuous variables that were conformed to normal distribution, and Mann–Whitney U-test was conducted for the skewed distribution data. Spearman correlation matrix was produced to calculate correlations between all continuous variables. The extreme values were regarded as missing data; then, all the missing data were interpolated using multiple imputation (mi) method. Next, using the predictors above, we constructed five machine learning prediction models: (1) logistic regression with lasso regularization (lasso regression), (2) Bayes logistic regression, (3) decision tree, (4) random forest, and (5) eXtreme Gradient Boosting (XGBoost). Logistic regression with lasso regularization could help shrink the regression coefficients toward zero but reserve the variables more stringent thresholds, which could finally build a parsimonious and predictive logistic regression model. The naïve Bayes method was conducted by using the Bayes’ theorem to evaluate post-error probabilities. A decision tree is a non-parametric model and a tree-like graph, which could easily establish classification rules, interpretation, and good interpretability; CART was conducted as decision tree algorithm. For random forest and XGBoost, different combinations (top N) of features were tested, and a 20-fold cross-validation was calculated. When the number of negative events was much greater than that of positive events, PRC (precision recall curve) could measure the quality of protective model more effectively and provide more information than area under the receiver operating characteristic (AUC-ROC) curve (15). In this study, AUC-PRC, accuracy, sensibility, specificity, positive predictive value (PPV), and negative predictive value (NPV) from the confusion matrix were conducted to measure each model. Statistical analyses were performed using the StataSE 15.1 (Stata Corporation, College Station, Texas), software R version 4.0.2 (Vienna, Austria), and the tidymodels packages. *P*-value was considered statistically significant below the 5% level.

## 3 Results

### 3.1 Summary of the training and validation sets

A total of 5,727 patients collected from MIMIC-IV database, 815 patients collected from eICU-CRD database, and 459 patients collected from dtChina database were finally enrolled in our study (**Figure 1**). The baseline characteristics, clinical tests, interventions, and in-hospital mortality of the study cohorts are shown in **Table 1**. There was no significant difference between the training set and the internal validation set. Among all the patients, the mortality rates of the training set, internal validation set, external validation set 1, and external validation set 2 were 12.70%, 12.69%, 12.27%, and 15.03%, respectively. In addition, there were no significant differences of in-hospital mortality between each dataset (**Table S1**, see **Supplementary Materials**; *P* = 0.315), and the differences in the clinical characteristics between hospitalized dead patients and non-dead patients in different databases are shown in **Tables S2–S4** (see **Supplementary Materials**).

**Table 1:** Baseline characteristics, clinical tests, interventions, and outcomes of the study cohorts.

Variable	MIMIC-IV database			eICU-CRD dataset	dtChina dataset
	Training set (n = 3780)	Internal validation set (n = 1947)	*P*-value	Externalvalidation set 1(n = 815)	External validation set 2 (n = 459)
**Age (Mean ± SD)**	67.85 ± 13.27	67.65 ± 13.50	0.587	66.35 ± 13.47	71.06 ± 14.16
**Gender male, n (%)**	2235 (59.13)	1132 (58.14)	0.473	408 (50.06)	251 (54.68)
**Comorbidity**					
Myocardial infarction, n (%)	952 (25.19)	505 (25.94)	0.536	67 (8.22)	60 (13.07)
Congestive heart failure, n (%)	1526 (40.37)	814 (41.81)	0.294	187 (22.94)	92 (20.04)
Peripheral vascular disease, n (%)	545 (14.42)	281 (14.43)	0.988	14 (1.71)	43 (9.37)
Cerebrovascular disease, n (%)	540 (14.29)	265 (13.61)	0.486	43 (5.28)	171 (37.25)
Dementia, n (%)	217 (5.74)	117 (6.01)	0.681	30 (3.68)	4 (0.87)
Chronic pulmonary disease, n (%)	1103 (29.18)	538 (27.63)	0.220	156 (19.14)	99 (21.57)
Peptic ulcer disease, n (%)	113 (2.99)	69 (3.53)	0.257	6 (0.74)	39 (8.50)
Paraplegia, n (%)	169 (4.47)	86 (4.42)	0.925	2 (0.25)	2 (0.44)
Renal disease, n (%)	1449 (38.33)	763 (39.19)	0.529	68 (8.34)	36 (7.84)
Malignant cancer, n (%)	493 (13.04)	248 (13.74)	0.745	16 (1.96)	10 (2.18)
Metastatic solid tumor, n (%)	177 (4.68)	93 (4.78)	0.874	4 (0.49)	29 (6.32)
**Vital signs**					
Heart rate (/min)	86.48 ± 15.76	86.47 ± 16.10	0.985	92.03 ± 22.00	105.28 ± 26.26
Systolic blood pressure (mmHg)	117.78 ± 15.99	117.45 ± 16.11	0.468	120.59 ± 24.80	138.26 ± 39.22
Diastolic blood pressure (mmHg)	61.23 ± 10.59	61.00 ± 10.30	0.439	62.94 ± 16.81	78.37 ± 23.98
Mean blood pressure (mmHg)	76.98 ± 10.37	76.62 ± 10.26	0.217	79.16 ± 18.08	98.13 ± 27.46
Respiratory rate (/min)	19.71 ± 3.90	19.72 ± 3.81	0.961	20.76 ± 6.66	21.72 ± 7.11
O_2_ saturation (%)	96.90 ± 2.29	96.89 ± 2.21	0.883	96.41 ± 4.12	91.87 ± 10.15
**Laboratory tests**					
White blood cell (K/µl)	13.54 ± 11.79	13.08 ± 8.25	0.127	13.15 ± 7.33	12.80 ± 6.10
Neutrophils (K/µl)	12.15 ± 11.09	11.76 ± 8.03	0.179	10.61 ± 6.50	11.10 ± 5.74
Lymphocytes (K/µl)	1.14 ± 4.24	1.18 ± 2.69	0.679	1.33 ± 1.34	1.14 ± 1.08
Monocytes (K/µl)	0.33 ± 0.84	0.33 ± 0.80	0.901	0.85 ± 0.78	0.55 ± 0.38
Eosinophils (K/µl)	0.13 ± 0.29	0.12 ± 0.17	0.240	0.13 ± 0.20	0.09 ± 0.15
Basophils (K/µl)	0.03 ± 0.06	0.03 ± 0.03	0.388	0.10 ± 0.21	0.03 ± 0.03
Red blood cell (m/µl)	3.47 ± 0.76	3.42 ± 0.78	0.018	3.65 ± 0.75	3.79 ± 0.90
Platelet (K/µl)	201.61 ± 107.94	204.12 ± 114.88	0.415	233.16 ± 113.46	179.38 ± 107.48
Hematocrit (%)	31.33 ± 6.44	30.94 ± 6.54	0.029	32.34 ± 6.55	33.51 ± 9.45
Hemoglobin (g/ml)	10.18 ± 2.14	10.01 ± 2.20	0.006	10.57 ± 2.13	11.24 ± 2.94
MCH (pg)	29.51 ± 2.80	29.45 ± 2.93	0.477	29.10 ± 2.68	29.71 ± 3.09
MCHC (g/ml)	32.48 ± 1.73	32.34 ± 1.75	0.005	32.61 ± 1.67	32.25 ± 1.81
MCV (fl)	90.91 ± 7.57	91.09 ± 7.61	0.391	89.12 ± 7.04	92.06 ± 7.73
RDW (%)	15.41 ± 2.40	15.53 ± 2.42	0.070	15.90 ± 2.47	14.44 ± 1.91
ALT (U/L)	120.34 ± 416.67	107.12 ± 354.94	0.232	86.37 ± 239.74	57.03 ± 155.89
AST (U/L)	213.73 ± 875.44	178.43 ± 737.16	0.128	107.81 ± 279.54	98.32 ± 383.41
ALP (U/L)	120.08 ± 110.23	121.76 ± 113.46	0.590	121.89 ± 95.09	105.09 ± 88.91
Albumin (g/ml)	3.35 ± 0.68	3.36 ± 0.68	0.851	3.36 ± 1.73	3.28 ± 0.69
Total bilirubin (mg/dl)	1.32 ± 2.98	1.30 ± 2.77	0.843	0.92 ± 0.94	1.43 ± 3.10
BUN (mg/dl)	35.38 ± 23.21	34.63 ± 23.41	0.248	36.51 ± 24.79	34.42 ± 26.66
Creatinine (mg/dl)	2.00 ± 1.79	2.00 ± 2.04	0.936	2.45 ± 2.06	1.70 ± 1.77
PT (s)	17.81 ± 9.74	18.30 ± 23.71	0.266	19.40 ± 11.06	15.04 ± 5.63
INR	1.63 ± 0.95	1.63 ± 0.91	0.855	1.78 ± 1.29	1.30 ± 0.54
Sodium (mmol/L)	138.08 ± 4.97	137.70 ± 4.75	0.005	136.99 ± 6.54	137.81 ± 5.97
Potassium (mmol/L)	4.47 ± 0.68	4.47 ± 0.67	0.711	4.44 ± 0.91	3.95 ± 0.90
Calcium (mg/dl)	8.42 ± 0.90	8.41 ± 0.82	0.941	8.74 ± 0.91	8.83 ± 0.99
Chloride (mmol/L)	102.61 ± 6.06	102.36 ± 6.04	0.150	100.15 ± 7.64	101.95 ± 6.65
Bicarbonate (mmol/L)	22.43 ± 4.41	22.34 ± 4.42	0.473	24.20 ± 6.38	20.23 ± 6.30
Anion gap	16.19 ± 4.24	16.06 ± 4.19	0.277	11.48 ± 5.12	15.62 ± 6.34
Glucose (mg/dl)	191.41 ± 103.25	189.09 ± 89.32	0.400	206.43 ± 150.61	273.95 ± 182.38
Lactate (mmol/L)	2.53 ± 2.17	2.54 ± 2.10	0.962	2.62 ± 1.99	3.71 ± 3.77
**Intervention**					
Vasopressor used, n (%)	1850 (48.94)	985 (50.59)	0.237	150 (18.40)	163 (35.51)
CRRT, n (%)	427 (11.30)	241 (12.38)	0.227	45 (5.52)	23 (5.01)
Ventilation, n (%)	1934 (51.17)	1014 (52.08)	0.511	300 (36.81)	326 (71.02)
**In-hospital mortality**, n (%)	480 (12.70)	247 (12.69)	0.989	100 (12.27)	69 (15.03)

### 3.2 Model construction and validation

The correlation of the continuous variable presented a good data consistency between each database (**Figure S1**, see **Supplementary Materials**). Then, logistic regression with lasso regularization, Bayes logistic regression, decision tree, random forest, and XGBoost were conducted to build the predictive model by using training set, whereas the internal and external validation sets were used for estimating the generalization capability of each model.

#### 3.2.1 Logistic regression with lasso regularization

**Figure 2:**
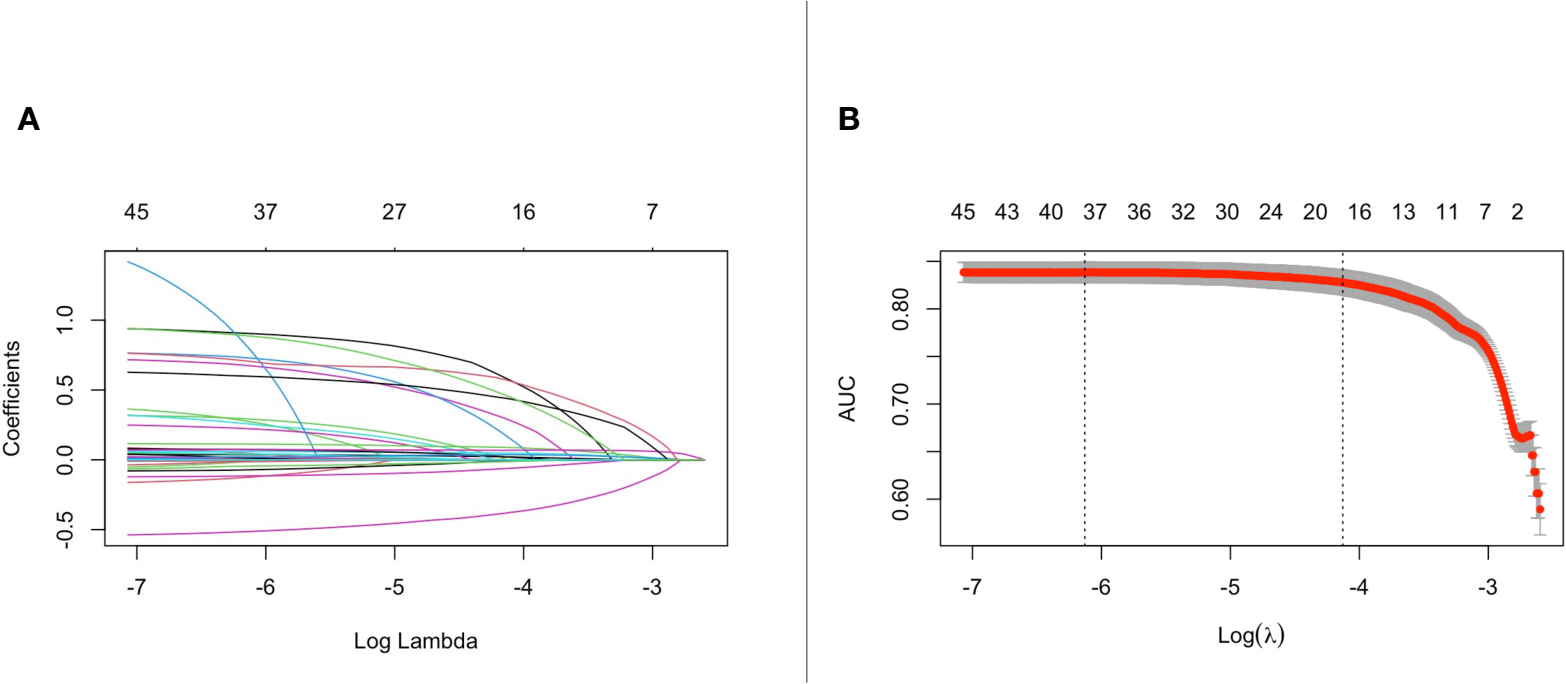
The change of the coefficient of different variables with penalty parameter λ set **(A)**. Penalty parameter plot **(B)**.

**Figure 3:**
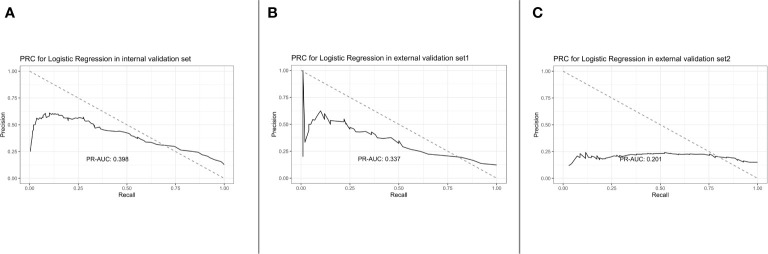
PRC for logistic regression in the internal validation set, external validation set 1, and external validation set 2 [from **(A)** to **(C)**].

After applying the lasso regularization, 38 variables were finally used for logistic regression analysis (**Figure 2**). There were 17 independent risk factors: age, comorbidity of myocardial infarction, cerebrovascular disease, renal disease, metastatic solid tumor, heart rate, respiratory rate, O_2_ saturation, MCHC, RDW, albumin, total bilirubin, bicarbonate, lactate, vasopressor use, CRRT, and ventilation (**Table 2**). Moreover, for the internal validation set, this model obtained a PR-AUC = 0.398, accuracy = 0.878, sensitivity = 0.896, specificity = 0.551, PPV = 0.974, and NPV = 0.219. For the external validation set 1, this model obtained a PR-AUC = 0.337, accuracy = 0.878, sensitivity = 0.883, specificity = 0.546, PPV = 0.993, and NPV = 0.060. For the external validation set 2, this model obtained a PR-AUC = 0.201, accuracy = 0.715, sensitivity = 0.863, specificity = 0.196, PPV = 0.790, and NPV = 0.290 (**Figure 3**).

**Table 2:** Multivariate logistic regression analysis with lasso regularization of in-hospital mortality in the training set.

Risk factors	Z	SE	OR	95% CI	P-value
Age (years)	7.821	0.005	1.041	1.030–1.052	<0.001
Myocardial infarction (yes vs. no)	2.613	0.125	1.388	1.084–1.773	0.009
Cerebrovascular disease (yes vs. no)	6.220	0.147	2.498	1.868–3.327	<0.001
Renal disease (yes vs. no)	2.149	0.134	1.334	1.025–1.734	0.032
Metastatic solid tumor (yes vs. no)	4.172	0.234	2.654	1.672–4.188	<0.001
Heart rate (/min)	3.183	0.004	1.012	1.005–1.019	0.001
Respiratory rate (/min)	3.781	0.016	1.062	1.029–1.096	<0.001
O_2_ saturation (%)	−5.251	0.026	0.873	0.829–0.918	<0.001
MCHC (g/ml)	−2.030	0.039	0.925	0.857–0.997	0.042
RDW (%)	4.210	0.025	1.109	1.057–1.164	<0.001
Albumin (g/ml)	−5.525	0.093	0.599	0.500–0.718	<0.001
Total bilirubin (mg/dl)	4.725	0.018	1.091	1.052–1.131	<0.001
Bicarbonate (mmol/L)	−2.432	0.016	0.962	0.933–0.992	0.015
Lactate (mmol/L)	3.018	0.026	1.081	1.027–1.137	0.003
Vasopressor use (yes vs. no)	4.215	0.145	1.842	1.389–2.453	<0.001
CRRT (yes vs. no)	4.737	0.158	2.116	1.550–2.883	<0.001
Ventilation (yes vs. no)	7.040	0.138	2.640	2.020–3.469	<0.001

#### 3.2.2 Bayes logistic regression

**Figure 4:**
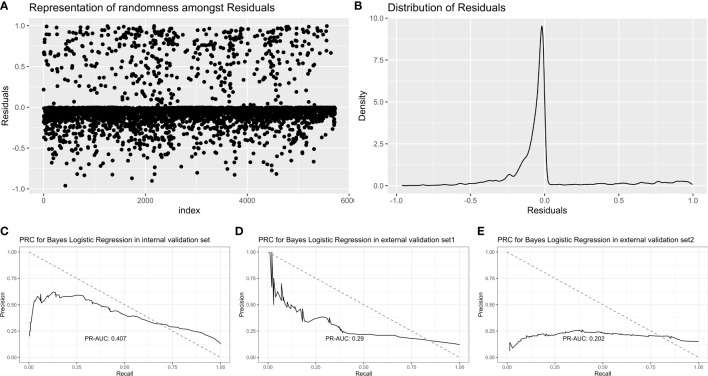
Representation of randomness among residuals **(A)**. Distribution of residuals **(B)**. PR-AUC for Bayes logistic regression in the internal validation set, the external validation set 1, and the external validation set 2 [from **(C–E)**].

Bayes logistic regression was conducted as described by a previous study (16). The randomness and distribution of all variables are shown in **Figures 4A, B**. For the internal validation set, the Bayes logistic regression model obtained a PR-AUC = 0.407, accuracy = 0.883, sensitivity = 0.900, specificity = 0.591, PPV = 0.975, and NPV = 0.251. For the external validation set 1, this model obtained a PR-AUC = 0.290, accuracy = 0.877, sensitivity = 0.888, specificity = 0.500, PPV = 0.983, and NPV = 0.120. For the external validation set 2, this model obtained a PR-AUC = 0.202, accuracy = 0.745, sensitivity = 0.874, specificity = 0.245, PPV = 0.818, and NPV = 0.333 (**Figures 4C–E**).

#### 3.2.3 Decision tree

**Figure 5:**
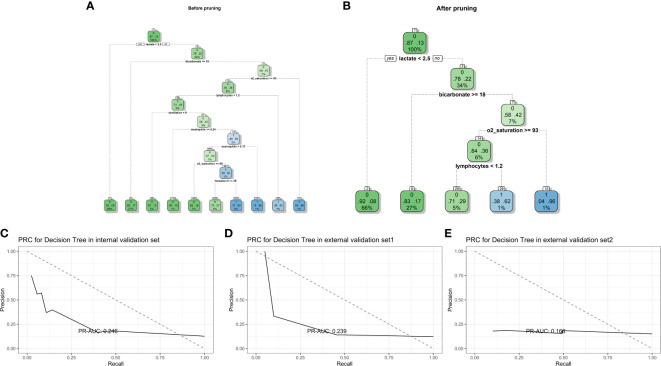
Decision tree model diagram before and after pruning **(A, B)**. PR-AUC for decision tree in the internal validation set, the external validation set 1, and the external validation set 2 [from **(C–E)**].

The decision tree model was established using all potential risk factors. After pruning, four influencing factors with five depth and five leaf nodes were found in our study: lymphocytes, oxygen saturation, bicarbonate level, and lactate level (**Figures 5A, B**). For the internal validation set, the CART decision tree model obtained a PR-AUC = 0.246, accuracy = 0.865, sensitivity = 0.984, specificity = 0.049, PPV = 0.877, and NPV = 0.300. For the external validation set 1, this model obtained a PR-AUC = 0.239, accuracy = 0.865, sensitivity = 0.972, specificity = 0.100, PPV = 0.885, and NPV = 0.333. For the external validation set 2, this model obtained a PR-AUC = 0.159, accuracy = 0.763 sensitivity = 0.867, specificity = 0.174, PPV = 0.856, and NPV = 0.187 (**Figures 5C–E**).

#### 3.2.4 Random forest

**Figure 6:**
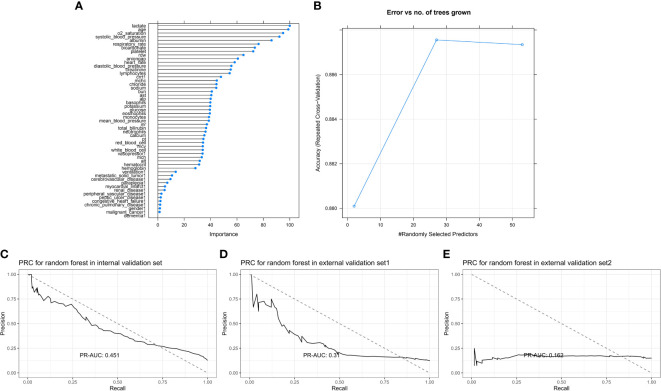
Feature importance score plot of random forest **(A)**. Model accuracy of random forest influenced by different number of randomly selected predictors **(B)**. PR-AUC for random forest in the internal validation set, the external validation set 1, and the external validation set 2 [from **(C–E)**].

A total of 53 indicators were tested, a 20-fold cross-validation was calculated, and the importance and number of errors are shown in **Figures 6A, B**. The five most predictive variables were lactate level, age, oxygen saturation at admission, systolic blood pressure at admission, and albumin level. For the internal validation set, the random forest model obtained a PR-AUC = 0.451, accuracy = 0.882, sensitivity = 0.992, specificity = 0.126, PPV = 0.887, and NPV = 0.705. For the external validation set 1, this model obtained a PR-AUC = 0.310, accuracy = 0.886, sensitivity = 0.989, specificity = 0.150, PPV = 0.893, and NPV = 0.652. For the external validation set 2, this model obtained a PR-AUC = 0.162, accuracy = 0.760 sensitivity = 0.874, specificity = 0.116, PPV = 0.848, and NPV = 0.140 (**Figures 6C–E**).

#### 3.2.5 XGBoost

**Figure 7:**
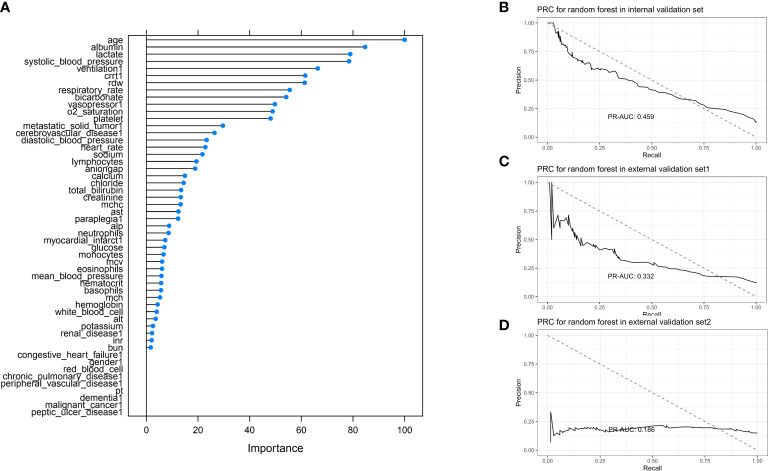
Feature importance score plot of XGBoost **(A)**. Model accuracy of XGBoost influenced by different number of randomly selected predictors **(B)**. PR-AUC for XGBoost in the internal validation set, the external validation set 1, and the external validation set 2 [from **(C, D)**].

XGBoost model was conducted by using a 20-fold cross-validation, and the top five most predictive variables were age, albumin level, lactate, systolic blood pressure, and ventilation (**Figure 7A**). For the internal validation set, the random forest model obtained a PR-AUC = 0.459, accuracy = 0.882, sensitivity = 0.978, specificity = 0.227, PPV = 0.897, and NPV = 0.596. For the external validation set 1, this model obtained a PR-AUC = 0.332, accuracy = 0.875, sensitivity = 0.971, specificity = 0.190, PPV = 0.896, and NPV = 0.475. For the external validation set 2, this model obtained a PR-AUC = 0.186, accuracy = 0.699 sensitivity = 0.777, specificity = 0.261, PPV = 0.856, and NPV = 0.171 (**Figures 7B–D**).

### 3.3 Models’ comparison

**Figure 8:**
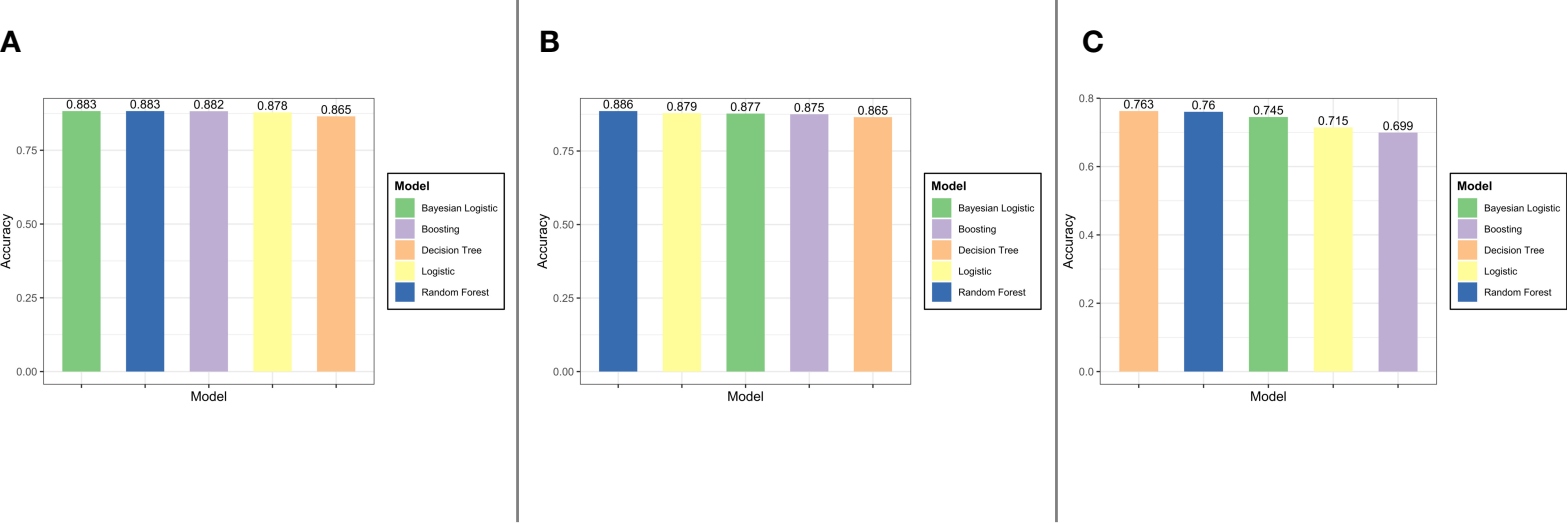
The result of models performance in the internal validation set, the external validation set 1, and the external validation set 2 (from **(A–C)**].

The results of model performance in predicting in-hospital mortality for the sepsis patients with diabetes are shown in **Figure 8**. The top three models for internal validation set were Bayes logistic regression, random forest, and XGBoost, whereas the top three models for the external validation set 1 were random forest, logistic regression, and Bayes logistic regression. In addition, the top three models for the external validation set 2 were decision tree, random forest, and Bayes logistic regression.

## 4 Discussion

Both ROC curves and PR curves provide a diagnostic tool to evaluate the performance of binary classification models. ROC curves visualize the trade-off between the true positive rate (TPR) and the false positive rate (FPR). However, PR-AUC focuses on precision (PPV) and recall (TPR) (17). Because of this, although there is some disturbance of the proportion of positive and negative samples in the test set changes, the ROC curve can remain unchanged (18). This consistency of the ROC in the face of class imbalance reflects its ability to measure the predictive performance of a model itself, which is independent of the proportion of the positive and negative samples. Otherwise, compared with ROC curves, PRC curves retain the sensibility to the change of proportion (15, 19–21). Hence, in case of imbalance between the positive and negative sample ratios, PRC is more responsive to the goodness of the classifier than ROC. It is the reason why, in this research, we use PR-AUC to present our results.

We analyzed a dataset composed of clinical data from 7,001 patients in the East and the West. Gender ratio in this present study is 3:4 (female:male), and the ratio does not show appreciable differences among different datasets. On the other hand, it seems like there is no relationship between gender and outcome, according to the results of Pearson’s chi-squared test with Yates’ continuity correction. The average age is 67.8 ± 13.4, and, according to not only the two feature importance score plots but also the rest predictor, age is a statistically crucial predictor variable of decease. For the elder people, even if without underlying diseases, sepsis is a critical health issue and a major cause of admission to ICU (22). Furthermore, older patients suffer longer length of stay in ICU and higher case fatality rates (23). More prolonged proinflammatory responses may count for much. Aging, on the other hand, is one of the nonmodifiable factors that contribute to the increased incidence of diabetes. It is suggested that aging and lower vascular telomere length in patients with Type 2 Diabetes (24) converge to endothelial dysfunction, which is an indicator of sepsis severity.

Lactate, catalyzed by lactate dehydrogenase, is a product of glucose metabolism. In our research, it is one of the most important variables of in-hospital mortality prediction for patients with sepsis with diabetes. In clinical use, the serum level of lactate is commonly included in management of patients with sepsis because of the impaired pyruvate dehydrogenase in sepsis. According to the sepsis-3 guidelines, septic shock should be clinically defined in case of persistence of a serum lactate more than 2 mmol/L despite adequate fluid resuscitation (1). The high lactate concentrations are suggested to be a predictor of mortality. Meanwhile, the lower lactate levels are related to improved clinical outcomes (25). Mechanically, some research studies indicated that the extracellular lactate may have important regulatory effects on a variety of immune cells (26). There is view that aerobic glycolytic metabolism is important to initiate immune cells (27). Compared with non-diabetic ones, the patients with diabetes have higher plasma lactate levels even in the prediabetes stage and hyperinsulinemia condition. Intriguingly, there is some evidence to indicate that lactate can be used to predict the occurrence of diabetes (28, 29). However, there is no research study that reveals direct association between sepsis and diabetes *via* lactate, especially for lactate, in metabolism disorder; a question remained: cause or consequence or both?

In this study, all the predictors are performed on the basis of the albumin levels. Classically, albumin levels reflect the nutritional status and organic function of patient. A research revealed that patients with low albumin had higher mortality and longer length of hospital stays than patients with normal albumin, whereas patients with high albumin had lower mortality and shorter hospital stays (30). Intriguingly, a prospective observational study concluded that hypoalbuminemia during sepsis was caused by enhanced clearance from the circulation instead of dysfunction of the liver (31). Moreover, ischemia modified albumin cloud be an effective diagnosis marker of neonatal sepsis (32).

Holistically, we noticed the performance level in the Chinese validation dataset decreased. Owing to the limit to the size of accessible matched dataset, our attempt to train the predictor with Chinese data and to use Western data for validation failed. Although, the racial differences should be considered. There are some published research studies that reveal the increased incidence and severity of sepsis in black individuals compared with that in white individuals (33–35). Furthermore, an excellent research proved that APOL1 risk variants, which are specifically present in individuals of African ancestry, contribute to the exacerbated sepsis (36). From this point, the conformance of responses to sepsis between different races should not be expected.

## 5 Conclusion

To predict the outcomes of patients with sepsis with diabetes, five machine learning models were established and validated. Random forest model performed well with the training and three validation sets. Among all variables of data, age, lactate, and albumin could be of high diagnostic value. Our results provide an approach of applying of algorithms to resolve the issue about prediction of complicated disease conditions.

## Data availability statement

The original contributions presented in the study are included in the article/**Supplementary Material**. Further inquiries can be directed to the corresponding author.

## Ethics statement

This study was approved by the Institutional Review Broad (IRB) of the Massachusetts Institute of Technology (MIT) online (Record ID: 38889441), and informed consent was waived.

## Author contributions

CS contributed to the conception of the study. JQ and JL contributed significantly to analysis and manuscript preparation. HL helped perform the analysis with constructive discussions. KZ, ZD, NL, and DH helped records enrolled. All authors contributed to the article and approved the submitted version.

## Funding

This study was supported by the Outstanding Youth Scientist Foundation of Hunan Province(No.2022JJ10099), the Wisdom Accumulation and Talent Cultivation Project of Central South University(No.YX202104) and Key Project of Hunan Provincial Science and Technology Innovation (No.2020SK1010).

## Conflict of interest

The authors declare that the research was conducted in the absence of any commercial or financial relationships that could be construed as a potential conflict of interest.

## Publisher’s note

All claims expressed in this article are solely those of the authors and do not necessarily represent those of their affiliated organizations, or those of the publisher, the editors and the reviewers. Any product that may be evaluated in this article, or claim that may be made by its manufacturer, is not guaranteed or endorsed by the publisher.
