# Supplementary material for: Machine learning models to predict in-hospital mortality in septic patients with diabetes
Source: Front Endocrinol (Lausanne). 2022 Nov 16;13:1034251. doi: 10.3389/fendo.2022.1034251 (PMC9709414; doi:10.3389/fendo.2022.1034251)
Supplement: Supplementary file 1 [file DataSheet_1.docx]

**Table S1. The comparison of in-hospital mortality in different datasets.**

| **Variable** | **MIMIC-IV dataset**  **(n=5727)** | **eICU-CRD dataset**  **(n=815)** | **dtChina dataset**  **(n=459)** | *χ^2^* | ***P*** |
| --- | --- | --- | --- | --- | --- |
| Alive, n (%) | 5000 (87.31) | 715 (87.73) | 390 (84.97) | 2.313 | 0.315 |
| Death, n (%) | 727 (12.69) | 100 (12.27) | 69 (15.03) |  |  |

**Table S2. Characterization of the MIMIC-IV dataset cohort.**

| Variable | Total  Patients | Survival  Group | Non-survival  Group | *P* value |
| --- | --- | --- | --- | --- |
|  | (n=5727) | (n=5000) | (n=727) |  |
| Age (Mean±SD) | 67.78±13.35 | 67.13±13.29 | 72.29±12.87 | <0.001 |
| Gender male, n (%) | 3367 (58.79) | 2932 (59.83) | 435 (58.67) | 0.541 |
| Comorbidity |  |  |  |  |
| Myocardial infarction, n (%) | 1457 (25.44) | 1200 (24.00) | 257 (35.35) | <0.001 |
| Congestive heart failure, n (%) | 2340 (40.86) | 1961 (39.22) | 379 (52.13) | <0.001 |
| Peripheral vascular disease, n (%) | 826 (14.42) | 700 (14.00) | 126 (17.33) | 0.017 |
| Cerebrovascular disease, n (%) | 805 (14.06) | 646 (12.92) | 159 (21.87) | <0.001 |
| Dementia, n (%) | 334 (5.83) | 280 (5.60) | 54 (7.43) | 0.049 |
| Chronic pulmonary disease, n (%) | 1641 (28.65) | 1444 (28.88) | 197 (27.10) | 0.321 |
| Peptic ulcer disease, n (%) | 182 (3.18) | 147 (2.94) | 35 (4.81) | 0.007 |
| Paraplegia, n (%) | 255 (4.45) | 199 (3.98) | 56 (7.70) | <0.001 |
| Renal disease, n (%) | 2212 (38.62) | 1863 (37.26) | 349 (48.01) | <0.001 |
| Malignant cancer, n (%) | 741(12.94) | 591 (11.82) | 150 (20.62) | <0.001 |
| Metastatic solid tumor, n (%) | 270 (4.71) | 190 (3.80) | 80 (11.00) | <0.001 |
| Vital signs |  |  |  |  |
| Heart rate (/min) | 86.48±15.87 | 85.91±15.44 | 90.39±18.14 | <0.001 |
| Systolic blood pressure (mmHg) | 117.67±16.03 | 118.38±15.74 | 112.71±17.10 | <0.001 |
| Diastolic blood pressure (mmHg) | 61.15±10.49 | 61.34±10.40 | 59.86±11.02 | <0.001 |
| Mean blood pressure (mmHg) | 76.86±10.33 | 77.14±10.23 | 74.88±10.80 | <0.001 |
| Respiratory rate (/min) | 19.71±3.86 | 19.46±3.71 | 21.43±4.42 | <0.001 |
| O2 saturation (%) | 96.90±2.27 | 97.00±1.95 | 96.19±3.73 | <0.001 |
| Laboratory tests |  |  |  |  |
| White blood cell (K/uL) | 13.38±10.72 | 13.21±10.74 | 14.56±10.52 | 0.002 |
| Neutrophils (K/uL) | 11.75±10.44 | 11.57±10.41 | 13.00±10.57 | <0.001 |
| Lymphocytes (K/uL) | 1.15±3.78 | 1.16±3.98 | 1.09±1.97 | 0.628 |
| Monocytes (K/uL) | 0.33±0.82 | 0.33±0.86 | 0.30±0.52 | 0.233 |
| Eosinophils (K/uL) | 0.13±0.25 | 0.12±0.25 | 0.15±0.25 | 0.013 |
| Basophils (K/uL) | 0.03±0.05 | 0.03±0.05 | 0.03±0.04 | 0.383 |
| Red blood cell (m/uL) | 3.46±0.77 | 3.46±0.76 | 3.42±0.83 | 0.184 |
| Platelet (K/uL) | 202.46±110.35 | 203.13±108.96 | 197.92±119.42 | 0.234 |
| Hematocrit (%) | 31.20±6.48 | 31.17±6.42 | 31.36±6.85 | 0.462 |
| Hemoglobin (g/mL) | 10.12±2.16 | 10.14±2.14 | 9.99±2.29 | 0.074 |
| MCH (pg) | 29.49±2.85 | 29.49±2.82 | 29.46±2.99 | 0.749 |
| MCHC (g/mL) | 32.43±1.74 | 32.52±1.71 | 31.85±1.81 | <0.001 |
| MCV (fL) | 90.97±7.58 | 90.74±7.43 | 92.57±8.37 | <0.001 |
| RDW (%) | 15.45±2.41 | 15.30±2.30 | 16.50±2.81 | <0.001 |
| ALT (U/L) | 115.85±396.78 | 105.73±366.93 | 185.44±555.95 | <0.001 |
| AST (U/L) | 201.73±831.12 | 174.36±724.60 | 389.99±1338.71 | <0.001 |
| ALP (U/L) | 120.65±111.33 | 117.77±107.63 | 140.44±132.48 | <0.001 |
| Albumin (g/mL) | 3.35±0.68 | 3.40±0.67 | 3.04±0.71 | <0.001 |
| Total bilirubin (mg/dL) | 1.31±2.91 | 1.22±2.72 | 1.90±3.93 | <0.001 |
| BUN (mg/dL) | 35.13±23.28 | 33.86±22.52 | 43.83±26.35 | <0.001 |
| Creatinine (mg/dL) | 2.00±1.88 | 1.95±1.88 | 2.34±1.80 | <0.001 |
| PT (sec) | 17.98±15.93 | 17.59±16.32 | 20.62±12.64 | <0.001 |
| INR | 1.63±0.94 | 1.59±0.88 | 1.90±1.22 | <0.001 |
| Sodium (mmol/L) | 137.95±4.90 | 137.94±4.74 | 138.01±5.89 | 0.733 |
| Potassium (mmol/L) | 4.47±0.68 | 4.46±0.66 | 4.56±0.77 | <0.001 |
| Calcium (mg/dL) | 8.42±0.88 | 8.42±0.85 | 8.40±1.02 | 0.554 |
| Chloride (mmol/L) | 102.52±6.05 | 102.67±5.88 | 101.54±7.02 | <0.001 |
| Bicarbonate (mmol/L) | 22.40±4.42 | 22.66±4.21 | 20.61±5.30 | <0.001 |
| Anion gap | 16.15±4.22 | 15.82±3.95 | 18.35±5.27 | <0.001 |
| Glucose (mg/dL) | 190.62±98.73 | 189.84±99.62 | 195.98±92.28 | 0.117 |
| Lactate (mmol/L) | 2.54±2.15 | 2.37±1.90 | 3.67±3.18 | <0.001 |
| Intervention |  |  |  |  |
| Vasopressor used, n (%) | 2892 (49.50) | 2685 (46.30) | 207 (71.53) | <0.001 |
| CRRT, n (%) | 668 (11.66) | 483 (9.66) | 185 (25.45) | <0.001 |
| Ventilation, n (%) | 2948 (51.48) | 2433 (48.66) | 515 (70.84) | <0.001 |

CHD, Coronary heart disease; COPD, Chronic Obstructive Pulmonary Disease; ARDS, Acute Respiratory Distress Syndrome; CRP, C-reactive protein; BNP, Brain Natriuretic Peptide;

*, 24 hours before extubation.

**Table S3. Characterization of the eICU-CRD dataset cohort.**

| Variable | Total  Patients | Survival  Group | Non-survival  Group | *P* value |
| --- | --- | --- | --- | --- |
|  | (n=815) | (n=715) | (n=100) |  |
| Age (Mean±SD) | 66.35±13.47 | 65.86±13.54 | 69.89±12.43 | 0.005 |
| Gender male, n (%) | 408 (50.06) | 355 (49.65) | 53 (53.00) | 0.530 |
| Comorbidity |  |  |  |  |
| Myocardial infarction, n (%) | 67 (8.22) | 61 (8.53) | 6 (6.00) | 0.388 |
| Congestive heart failure, n (%) | 187 (22.94) | 165 (23.08) | 22 (22.00) | 0.810 |
| Peripheral vascular disease, n (%) | 14 (1.72) | 12 (1.68) | 2 (2.00) | 0.817 |
| Cerebrovascular disease, n (%) | 43 (5.28) | 37 (5.17) | 6 (6.00) | 0.730 |
| Dementia, n (%) | 30 (3.68) | 22 (3.08) | 8 (8.00) | 0.014 |
| Chronic pulmonary disease, n (%) | 156 (19.14) | 134 (18.74) | 22 (22.00) | 0.438 |
| Peptic ulcer disease, n (%) | 6 (0.74) | 6 (0.84) | 0 (0.00) | 1.000 |
| Paraplegia, n (%) | 2 (0.25) | 2 (0.28) | 0 (0.00) | 1.000 |
| Renal disease, n (%) | 68 (8.34) | 58 (8.11) | 10 (10.00) | 0.522 |
| Malignant cancer, n (%) | 16(1.96) | 12 (1.68) | 4 (4.00) | 0.117 |
| Metastatic solid tumor, n (%) | 4 (0.49) | 3 (0.42) | 1 (1.00) | 0.408 |
| Vital signs |  |  |  |  |
| Heart rate (/min) | 92.03±22.00 | 91.52±21.20 | 95.71±26.89 | 0.074 |
| Systolic blood pressure (mmHg) | 120.59±24.80 | 121.88±24.84 | 111.39±22.60 | <0.001 |
| Diastolic blood pressure (mmHg) | 62.94±16.81 | 63.54±16.92 | 58.68±15.38 | 0.007 |
| Mean blood pressure (mmHg) | 79.16±18.08 | 79.99±18.12 | 73.23±16.68 | <0.001 |
| Respiratory rate (/min) | 20.77±6.66 | 20.50±6.47 | 22.71±7.60 | 0.002 |
| O2 saturation (%) | 96.41±4.13 | 96.57±3.87 | 95.26±5.52 | 0.003 |
| Laboratory tests |  |  |  |  |
| White blood cell (K/uL) | 13.15±7.33 | 12.91±7.02 | 14.89±9.09 | 0.011 |
| Neutrophils (K/uL) | 10.61±6.50 | 10.39±6.23 | 12.17±8.03 | 0.011 |
| Lymphocytes (K/uL) | 1.33±1.34 | 1.33±1.33 | 1.40±1.40 | 0.596 |
| Monocytes (K/uL) | 0.85±0.78 | 0.83±0.75 | 0.97±0.94 | 0.081 |
| Eosinophils (K/uL) | 0.13±0.20 | 0.14±0.20 | 0.09±0.14 | 0.056 |
| Basophils (K/uL) | 0.11±0.21 | 0.10±0.16 | 0.11±0.42 | 0.606 |
| Red blood cell (m/uL) | 3.65±0.75 | 3.65±0.75 | 3.59±0.72 | 0.461 |
| Platelet (K/uL) | 233.16±113.46 | 235.35±113.73 | 217.48±110.75 | 0.140 |
| Hematocrit (%) | 32.34±6.55 | 32.33±6.58 | 32.44±6.36 | 0.876 |
| Hemoglobin (g/mL) | 10.57±2.13 | 10.59±2.13 | 10.43±2.10 | 0.497 |
| MCH (pg) | 29.10±2.68 | 29.10±2.66 | 29.05±2.83 | 0.852 |
| MCHC (g/mL) | 32.61±1.67 | 32.66±1.65 | 32.21±1.77 | 0.010 |
| MCV (fL) | 89.12±7.04 | 88.96±6.98 | 90.25±7.35 | 0.087 |
| RDW (%) | 15.90±2.47 | 15.80±2.42 | 16.67±2.74 | 0.001 |
| ALT (U/L) | 86.37±239.74 | 82.98±244.79 | 110.62±199.39 | 0.281 |
| AST (U/L) | 107.81±279.54 | 96.50±264.64 | 264.64±360.34 | 0.002 |
| ALP (U/L) | 121.89±95.09 | 119.80±94.90 | 136.88±95.58 | 0.092 |
| Albumin (g/mL) | 3.36±1.73 | 3.37±1.71 | 3.26±1.82 | 0.532 |
| Total bilirubin (mg/dL) | 0.92±0.94 | 0.90±0.94 | 1.03±0.98 | 0.198 |
| BUN (mg/dL) | 36.51±24.79 | 36.24±24.58 | 38.49±26.34 | 0.396 |
| Creatinine (mg/dL) | 2.45±2.06 | 2.42±2.09 | 2.69±1.87 | 0.227 |
| PT (sec) | 19.40±11.06 | 19.14±10.51 | 21.22±14.34 | 0.080 |
| INR | 1.78±1.29 | 1.76±1.27 | 1.90±1.42 | 0.291 |
| Sodium (mmol/L) | 136.99±6.54 | 136.88±6.17 | 137.84±8.72 | 0.166 |
| Potassium (mmol/L) | 4.44±0.91 | 4.45±0.91 | 4.44±0.87 | 0.916 |
| Calcium (mg/dL) | 8.74±0.91 | 8.78±0.90 | 8.49±0.89 | 0.003 |
| Chloride (mmol/L) | 100.15±7.64 | 100.02±7.27 | 101.12±9.91 | 0.176 |
| Bicarbonate (mmol/L) | 24.20±6.38 | 24.33±6.32 | 23.23±6.78 | 0.105 |
| Anion gap | 11.48±5.12 | 11.27±4.85 | 13.05±6.56 | 0.001 |
| Glucose (mg/dL) | 206.43±150.61 | 207.98±155.55 | 195.34±108.91 | 0.432 |
| Lactate (mmol/L) | 2.62±1.99 | 2.57±1.92 | 2.98±2.40 | 0.057 |
| Intervention |  |  |  |  |
| Vasopressor used, n (%) | 150 (18.40) | 106 (14.83) | 44 (44.00) | <0.001 |
| CRRT, n (%) | 45 (5.52) | 40 (5.59) | 5 (5.00) | 0.807 |
| Ventilation, n (%) | 300 (36.81) | 245 (34.27) | 55 (55.00) | <0.001 |

CHD, Coronary heart disease; COPD, Chronic Obstructive Pulmonary Disease; ARDS, Acute Respiratory Distress Syndrome; CRP, C-reactive protein; BNP, Brain Natriuretic Peptide;

*, 24 hours before extubation.

**Table S4. Characterization of the dtCHINA dataset cohort.**

| Variable | Total  Patients | Survival  Group | Non-survival  Group | *P* value |
| --- | --- | --- | --- | --- |
|  | (n=459) | (n=390) | (n=69) |  |
| Age (Mean±SD) | 71.07±14.16 | 70.03±13.82 | 76.90±14.69 | <0.001 |
| Gender male, n (%) | 251 (54.68) | 207 (53.08) | 44 (63.77) | 0.100 |
| Comorbidity |  |  |  |  |
| Myocardial infarction, n (%) | 60 (13.07) | 48 (12.31) | 12 (17.39) | 0.248 |
| Congestive heart failure, n (%) | 92 (20.04) | 75 (19.23) | 17 (24.64) | 0.301 |
| Peripheral vascular disease, n (%) | 43 (9.37) | 39 (10.00) | 4 (5.79) | 0.269 |
| Cerebrovascular disease, n (%) | 171 (37.25) | 138 (35.38) | 33 (47.83) | 0.049 |
| Dementia, n (%) | 4 (0.87) | 3 (0.77) | 1 (1.45) | 0.480 |
| Chronic pulmonary disease, n (%) | 99 (21.57) | 80 (20.51) | 19 (27.54) | 0.191 |
| Peptic ulcer disease, n (%) | 39 (8.50) | 30 (7.69) | 9 (13.04) | 0.142 |
| Paraplegia, n (%) | 2 (0.44) | 2 (0.51) | 0 (0.00) | 1.000 |
| Renal disease, n (%) | 36 (7.84) | 26 (6.67) | 10 (14.49) | 0.026 |
| Malignant cancer, n (%) | 10(2.18) | 10 (2.56) | 0 (0.00) | 0.371 |
| Metastatic solid tumor, n (%) | 29 (6.32) | 26 (6.67) | 3 (4.35) | 0.466 |
| Vital signs |  |  |  |  |
| Heart rate (/min) | 105.28±26.26 | 105.19±26.59 | 105.78±24.49 | 0.864 |
| Systolic blood pressure (mmHg) | 138.26±39.22 | 137.57±38.89 | 142.21±41.12 | 0.366 |
| Diastolic blood pressure (mmHg) | 78.37±23.98 | 78.59±23.96 | 77.09±24.28 | 0.632 |
| Mean blood pressure (mmHg) | 97.07±29.31 | 97.44±28.90 | 94.99±31.68 | 0.523 |
| Respiratory rate (/min) | 21.73±7.11 | 21.59±7.07 | 22.49±7.31 | 0.334 |
| O2 saturation (%) | 92.29±10.58 | 92.49±10.71 | 91.14±9.83 | 0.327 |
| Laboratory tests |  |  |  |  |
| White blood cell (K/uL) | 12.80±6.10 | 12.86±6.23 | 12.43±5.33 | 0.586 |
| Neutrophils (K/uL) | 11.11±5.74 | 11.16±5.90 | 10.80±4.77 | 0.627 |
| Lymphocytes (K/uL) | 1.14±1.08 | 1.15±1.09 | 1.11±1.06 | 0.774 |
| Monocytes (K/uL) | 0.55±0.38 | 0.56±0.38 | 0.52±0.37 | 0.508 |
| Eosinophils (K/uL) | 0.09±0.15 | 0.09±0.15 | 0.09±0.13 | 0.917 |
| Basophils (K/uL) | 0.03±0.03 | 0.03±0.03 | 0.04±0.05 | 0.277 |
| Red blood cell (m/uL) | 3.79±0.90 | 3.81±0.92 | 3.69±0.82 | 0.311 |
| Platelet (K/uL) | 179.38±107.48 | 180.39±111.33 | 173.66±82.90 | 0.632 |
| Hematocrit (%) | 33.51±9.45 | 33.57±9.47 | 33.17±9.39 | 0.750 |
| Hemoglobin (g/mL) | 11.24±2.94 | 11.29±2.97 | 10.96±2.75 | 0.397 |
| MCH (pg) | 29.71±3.09 | 29.68±3.03 | 29.90±3.40 | 0.582 |
| MCHC (g/mL) | 32.25±1.81 | 32.27±1.80 | 32.16±1.91 | 0.651 |
| MCV (fL) | 92.06±7.73 | 91.92±7.73 | 92.89±7.74 | 0.336 |
| RDW (%) | 14.44±1.91 | 14.39±1.83 | 14.73±2.29 | 0.169 |
| ALT (U/L) | 57.03±155.89 | 58.93±165.16 | 46.26±86.53 | 0.534 |
| AST (U/L) | 98.32±383.41 | 103.75±413.15 | 67.59±111.93 | 0.471 |
| ALP (U/L) | 105.09±88.91 | 108.42±94.37 | 86.25±43.24 | 0.056 |
| Albumin (g/mL) | 3.28±0.69 | 3.26±0.69 | 3.34±0.66 | 0.400 |
| Total bilirubin (mg/dL) | 1.43±3.10 | 1.51±3.34 | 0.96±0.79 | 0.178 |
| BUN (mg/dL) | 34.42±26.66 | 33.92±26.65 | 37.24±26.73 | 0.342 |
| Creatinine (mg/dL) | 1.70±1.77 | 1.66±1.78 | 1.92±1.72 | 0.258 |
| PT (sec) | 15.04±5.63 | 15.02±5.72 | 15.14±5.19 | 0.865 |
| INR | 1.30±0.54 | 1.30±0.55 | 1.30±0.46 | 0.968 |
| Sodium (mmol/L) | 137.81±5.97 | 137.73±5.85 | 138.25±6.65 | 0.503 |
| Potassium (mmol/L) | 3.95±0.90 | 3.92±0.87 | 4.12±1.05 | 0.083 |
| Calcium (mg/dL) | 8.84±0.99 | 8.82±1.02 | 8.91±0.79 | 0.493 |
| Chloride (mmol/L) | 101.95±6.65 | 102.20±6.47 | 100.53±7.45 | 0.053 |
| Bicarbonate (mmol/L) | 20.23±6.30 | 20.05±6.19 | 21.28±6.84 | 0.133 |
| Anion gap | 15.62±6.34 | 15.47±6.09 | 16.44±7.64 | 0.245 |
| Glucose (mg/dL) | 273.95±182.38 | 272.42±181.31 | 282.56±189.47 | 0.671 |
| Lactate (mmol/L) | 3.71±3.77 | 3.69±3.62 | 3.83±4.53 | 0.783 |
| Intervention |  |  |  |  |
| Vasopressor used, n (%) | 163 (35.51) | 131 (33.59) | 32 (46.38) | 0.041 |
| CRRT, n (%) | 23 (5.01) | 17 (4.36) | 6 (8.70) | 0.128 |
| Ventilation, n (%) | 326 (71.02) | 271 (69.49) | 55 (79.71) | 0.084 |

CHD, Coronary heart disease; COPD, Chronic Obstructive Pulmonary Disease; ARDS, Acute Respiratory Distress Syndrome; CRP, C-reactive protein; BNP, Brain Natriuretic Peptide;

*, 24 hours before extubation.
