# Supplementary figures and images for: Machine learning models to predict in-hospital mortality in septic patients with diabetes
Source: Front Endocrinol (Lausanne). 2022 Nov 16;13:1034251. doi: 10.3389/fendo.2022.1034251 (PMC9709414; doi:10.3389/fendo.2022.1034251)

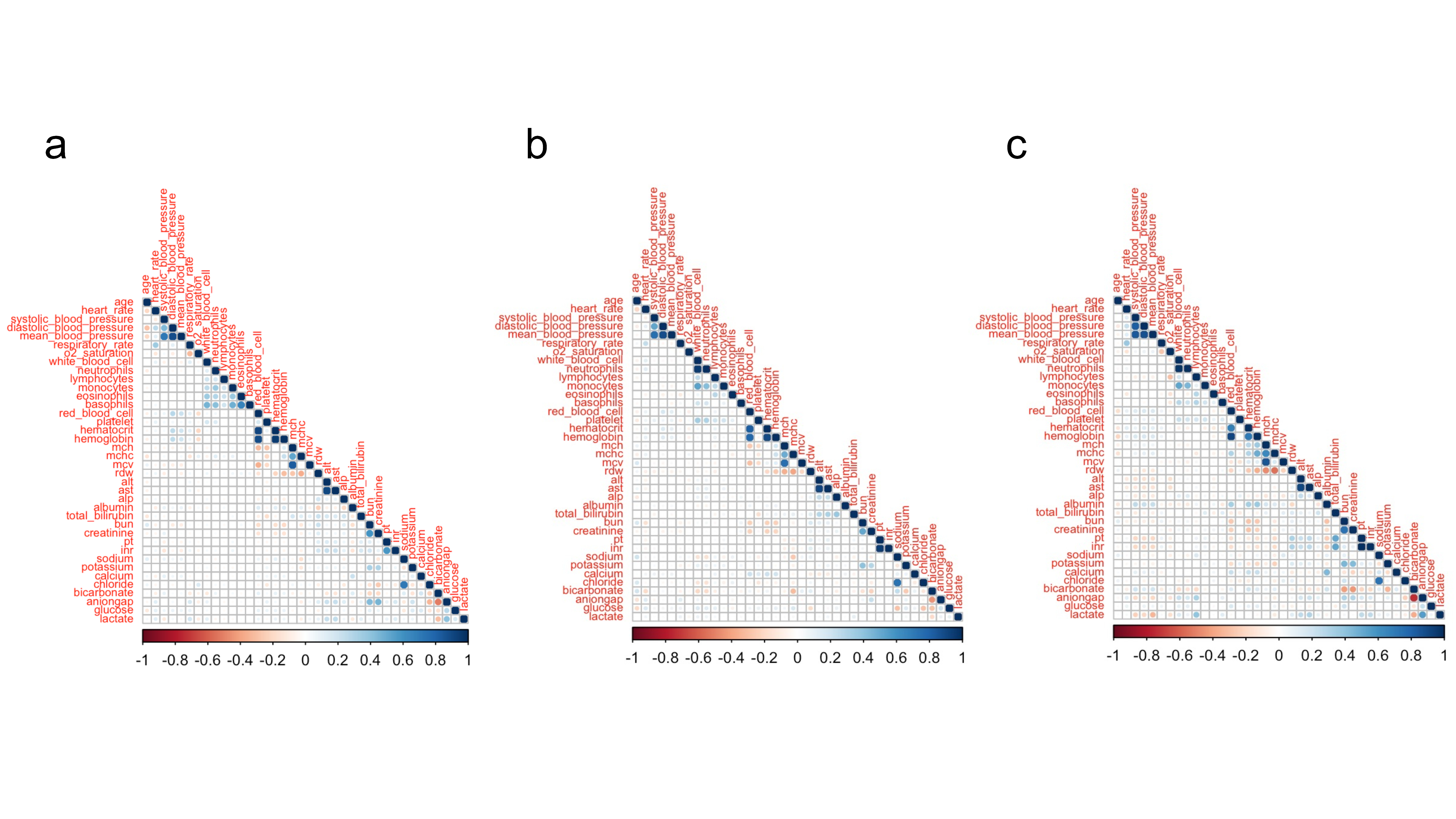

Supplement: Supplementary file 2 [file Image_1.jpeg]
